# 78. Aerobactin Gene iuc as a Virulence Marker Associated with Increased Mortality in Carbapenem-Resistant Klebsiella pneumoniae infections: A Multicenter Observational Cohort Study from China

**DOI:** 10.1093/ofid/ofaf695.029

**Published:** 2026-01-11

**Authors:** Jianping Jiang, Lauren Komarow, Yixuan Li, Angelique E Boutzoukas, Yunsong Yu, Yonghong Xiao, Hainv Gao, Zhengyin Liu, Vance G Fowler, Barry N Kreiswirth, Liang Chen, Minggui Wang, David van Duin

**Affiliations:** Center for Discovery and Innovation, Hackensack Meridian Health, Nutley, NJ; George Washington University, Rockville, Maryland; George Washington University, Rockville, Maryland; Duke University/Duke Clinical Research Institute, Raleigh, NC; Sir Run Run Shaw Hospital, Hangzhou, Zhejiang, China; State Key Laboratory for Diagnosis and Treatment of Infectious Diseases, The First Affiliated Hospital of Medical School of Zhejiang University, Hangzhou, Zhejiang, China; Shulan Hospital, Hangzhou, Zhejiang, China; Infectious Disease Section, Department of Internal Medicine, Peking Union Medical College Hospital, Beijing, Beijing, China; Duke University Medical Center, Durham, NC; Center for Discovery and Innovation, Hakensack Meridian Health, Nutley, NewJersey; SUNY-Buffalo, Buffalo, New York; Institute of Antibiotics, Huashan Hospital, Fudan University, Shanghai, Shanghai, China (People's Republic); University of North Carolina at Chapel Hill, Chapel Hill, NC

## Abstract

**Background:**

Despite growing reports of virulence gene acquisition in carbapenem-resistant *Klebsiella pneumoniae* (CRKP) strains, the clinical implications of these infections remain poorly understood.

**Figure:**
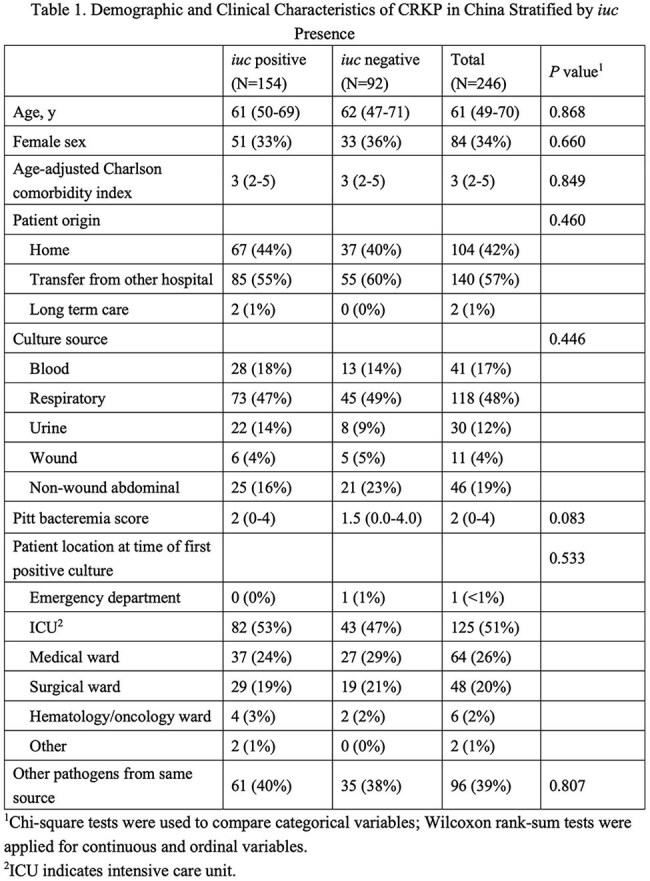

**Figure:**
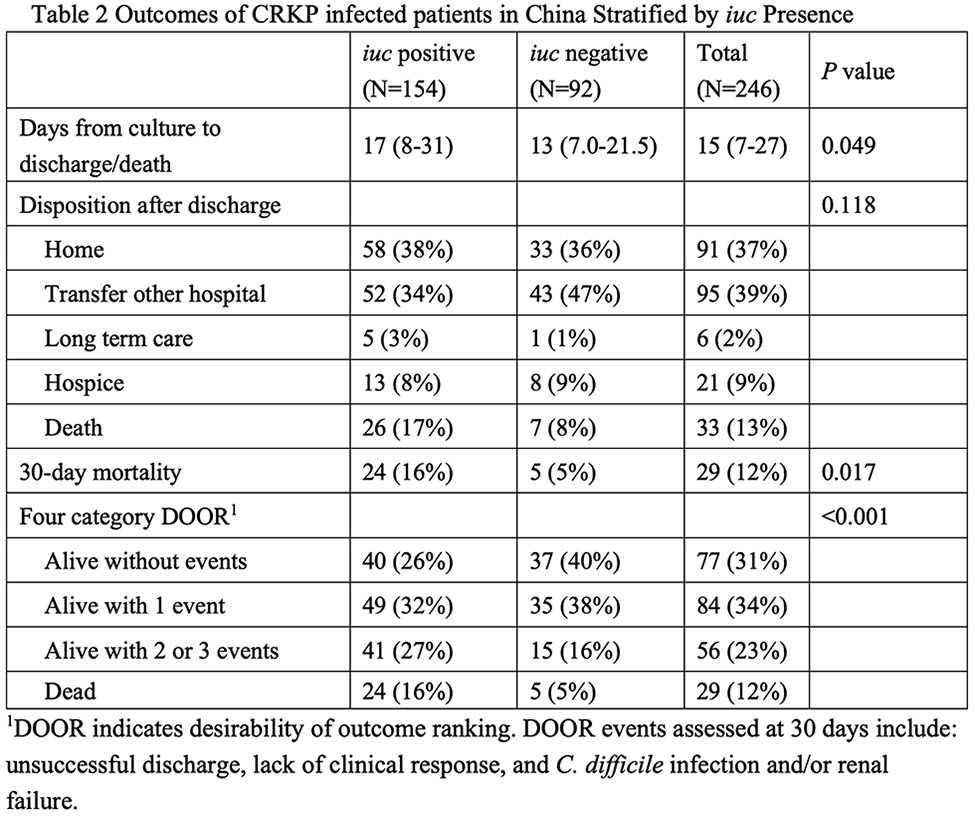

**Methods:**

In this study, 246 hospitalized patients with clinical CRKP infections were enrolled from six hospitals across China between 2017 and 2019 (CRACKLE-2 China Cohort). All CRKP isolates underwent whole-genome sequencing to determine sequence types and the presence of virulence genes, including *iuc* (aerobactin), *iro* (salmochelin), and mucoid phenotype regulators (*rmpADC* and *rmpA2*). Clinical, microbiological, and outcome data were compared by *iuc* status, and multivariable logistic regression was used to assess factors associated with 30-day mortality. Kaplan-Meier analysis was performed to evaluate 30-day survival.

**Figure:**
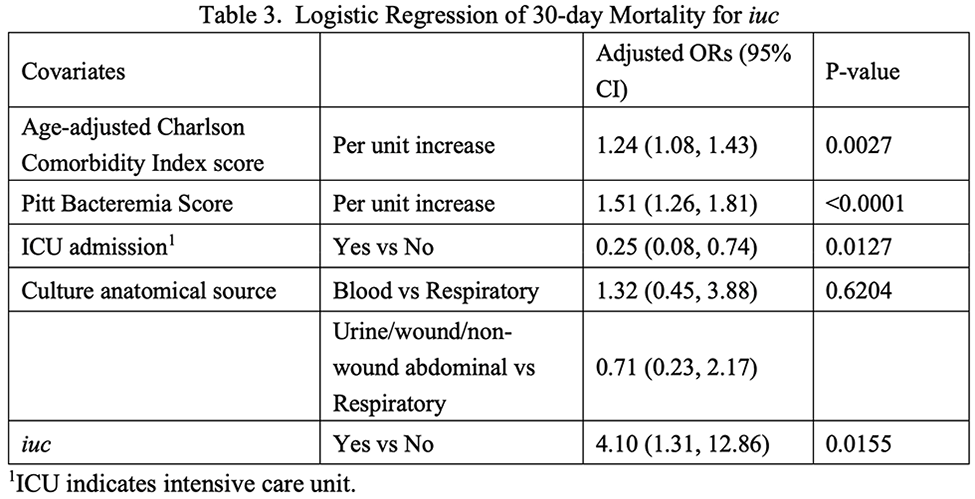

**Figure:**
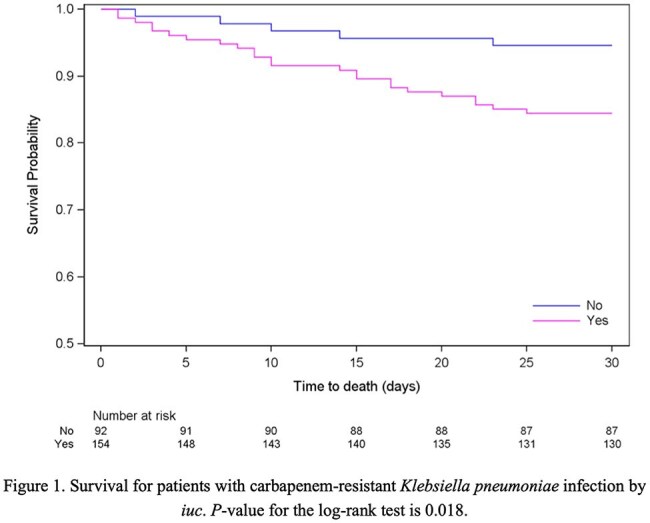

**Results:**

Among the 246 CRKP isolates, ST11 (78%, 192/246) and ST15 (14%, 34/246) were predominant. *iuc* was detected in 63% (154/246) of the isolates. *rmpA2* (Pearson coefficient *r*=0.9; *P*< 0.01) and *rmpA* (*r*=0.7; *P*< 0.01) were highly correlated with *iuc*. In only 6/246 isolates (2%) *iro* was detected, five of which were *iuc*-positive. Demographics and clinical characteristics did not differ between patients infected with *iuc*-positive and *iuc*-negative isolates (Table 1). Clinical outcomes were worse in *iuc*-positive CRKP infections; unadjusted 30-day mortality was higher in the *iuc*-positive group compared to the *iuc*-negative group (16% vs. 5%, 24/154 vs. 5/92; *P*< 0.05). Additionally, the 30-day DOOR outcomes were significantly different, and the presence of *iuc* was associated with less desirable outcomes (DOOR probability 37.8%; 95% Halperin confidence interval: 31.3%-44.7%, Table 2). After adjusting for potential confounders, *iuc*-positive CRKP infections remained independently predictive of increased 30-day all-cause mortality (adjusted odds ratio [aOR], 4.10; 95% CI, 1.31-12.86; *P*=0.016) (Table 3). Kaplan-Meier survival analysis further demonstrated significantly poorer survival among patients with *iuc*-positive infections (*log-rank p=0.018*) (Figure 1).

**Conclusion:**

These findings suggest that the presence of *iuc* contributes to increased mortality in CRKP infections and highlight the need for its surveillance in clinical settings.

**Disclosures:**

Angelique E. Boutzoukas, MD, MPH, Elion Therapeutics: Advisor/Consultant|Entasis Therapeutics Inc., an affiliate of Innoviva Specialty Therapeutics, Inc.: Board Member David van Duin, MD, PhD, AbbVie Inc: Advisor/Consultant|Merck & Co., Inc.: Advisor/Consultant|Merck & Co., Inc.: Grant/Research Support|Parexel International: DSMB|Pfizer, Inc.: Advisor/Consultant|Pfizer, Inc.: Honoraria|Roche Pharmaceuticals: Advisor/Consultant|TEVA: Advisor/Consultant

